# The impact of asymptomatic intracranial atherosclerotic stenosis on the clinical outcomes of patients with single subcortical infarction

**DOI:** 10.3389/fmed.2023.1249347

**Published:** 2023-09-01

**Authors:** Yi Yang, Yue He, Yuhao Xu, Wei Han, Yuanwei Shao, Tian Zhao, Ming Yu

**Affiliations:** ^1^Department of Neurology, Affiliated Hospital of Jiangsu University, Zhenjiang, China; ^2^Department of Radiology, Affiliated Hospital of Jiangsu University, Zhenjiang, China

**Keywords:** asymptomatic intracranial atherosclerotic stenosis, parental arterial disease, single subcortical infarction, post-stroke disability, predictor

## Abstract

**Background:**

The presence of parental arterial disease (PAD) is correlated with the outcomes of patients with a single subcortical infarction (SSI). Due to the relatively low incidence of PAD, the predictors of outcomes seem to be limited for SSI patients without PAD. This study aims to investigate the association between asymptomatic intracranial atherosclerotic stenosis (aIAS) and outcomes in patients with SSI and in the subgroup without PAD.

**Methods:**

Patients with SSI were consecutively enrolled. aIAS referred to a stenosis of ≥50% in intracranial arteries irrelevant to SSI by using magnetic resonance angiography. A poor outcome refers to a modified Ranking Scale >2 points at discharge.

**Results:**

In total, 298 participants were enrolled. The presence of aIAS could predict a poor outcome for all SSI patients [adjusted relative risk (aRR) = 2.14, 95% confidence interval (CI) = 1.17–3.93, *p* = 0.014] and in the subgroup without PAD (aRR = 3.12, 95% CI = 1.47–6.62, *p* = 0.003), but not in the subgroup with PAD. Compared with participants with neither aIAS nor PAD, the risk of a poor outcome increased approximately 2-fold in those with aIAS only (aRR = 2.95, 95% CI = 1.55–5.60, *p* = 0.001) and in those with concomitant aIAS and PAD (aRR = 3.10, 95% CI = 1.62–5.95, *p* = 0.001).

**Conclusion:**

The presence of aIAS is a predictor of a poor outcome in SSI patients, especially in those without PAD.

## 1. Introduction

Single subcortical infarction (SSI) accounts for 25–40% of patients with acute ischemic stroke (1, 2). Patients with SSI have a relatively high rate of early neurological deterioration (ND), which ranges from 20 to 43% (3–5). Due to the detrimental effect of ND on the clinical outcomes of patients with stroke, the early identification of predictive factors for outcomes in patients with SSI is essential.

SSI could be caused by two major vascular pathologies: (1) the orifice of a perforating artery being blocked by an atheroma of the parental artery and (2) lipohyalinosis or fibrinoid degeneration of the perforating arterial wall (6, 7). Given this specific pathogenic mechanism, parental arterial disease (PAD), i.e., any degree of stenosis on the parental artery (8, 9), could be considered the symptomatic stenosis of patients with SSI. The presence of PAD is closely correlated with stroke severity, the occurrence of ND, the functional outcome, and the recurrence of ischemic stroke in patients with SSI (1, 2, 8, 10, 11).

Despite the close associations between the presence of PAD and the prognoses of patients with SSI, the incidence of PAD accounts for only 15–35% of these patients (1, 2, 8, 10, 11). The majority of SSI patients lack PAD, and their infarctions probably result from the pathology of the perforating arterial wall. For these SSI patients without PAD, the predictors of clinical outcomes seem to be limited. A lot of previous studies have reported that intracranial significant stenosis of ≥50% could predict the clinical outcomes of patients with acute ischemic stroke (12–15). Furthermore, compared with the evaluation of a single vascular bed, the evaluation of atherosclerotic conditions in multiple vascular beds is likely to be better at predicting the clinical outcomes of patients with ischemic stroke (16). Therefore, we speculate that the evaluation of the atherosclerotic stenosis of other intracranial large arteries except the parental artery, namely the asymptomatic intracranial atherosclerotic stenosis (aIAS), could contribute to predicting the clinical outcomes of patients with SSI, especially for those without PAD. A recent study reported that patients with aIAS had a higher rate of vascular events than those without aIAS (17). Another study found that intracranial significant stenosis could compromise the perfusion in the basal ganglia and centrum ovale (18), which are the predilection sites of SSI, thus hampering the functional recovery of patients with SSI. These two studies might support our hypothesis.

We evaluated the presence of PAD and aIAS by using time-of-flight magnetic resonance angiography (MRA) in this single-center study, aiming to investigate the associations between the aIAS and the clinical outcomes in patients with SSI, especially in those without PAD. Given that SSI patients without PAD constitute the majority of the SSI population and are short of predictors of clinical outcomes, this study contributes to the early identification of these patients with a high risk of post-stroke disability and to strategize the administration of aIAS in these patients.

## 2. Materials and methods

### 2.1. Study population

This prospective single-center study was conducted according to the guidelines of the Declaration of Helsinki and reviewed and approved by the Research Ethics Committee of the Affiliated Hospital of Jiangsu University (protocol code: KY2023K0401). This study adhered to STrengthening the Reporting of OBservational studies in Epidemiology (STROBE) guidelines. We consecutively enrolled patients who were admitted to the stroke unit from 1 August 2021 to 31 August 2022. The inclusion criteria were (1) age ≥18 years; and (2) a definite single perforating infarct sited in the basal ganglia, corona radiata, internal capsule, or paramedian pontine area by using cranial magnetic resonance imaging (MRI). Because MRI is sensitive to patients' motions, a failure rate of 9.9% due to motion-related poor image quality was reported according to population-based studies (19, 20). Therefore, patients with poor image quality on MRI were not enrolled in the present study; and (3) admission within 7 days of symptom onset. Patients with potential sources of cardioembolism, e.g., atrial fibrillation, acute myocardial infarction, or cardiomyopathy with a pre-stroke modified Ranking Scale (mRS) >2 points were excluded. Because the pathological progression of non-atherosclerotic diseases, e.g., dissection, vasculitis, and Moyamoya disease, is likely to be different from that of atherosclerosis, the clinical outcomes of non-atherosclerotic diseases may differ from those of patients with atherosclerosis. Therefore, patients with the abovementioned non-atherosclerotic diseases were excluded from this study. Patients with severe stroke complications that might affect their daily functions at discharge, such as infection, electrolyte disorders, and epilepsy, were also excluded.

### 2.2. Baseline characteristics

According to the WHO criteria, patients with acute neurological deficits persisting for more than 24 h were diagnosed with acute ischemic stroke (21). On admission, patients' baseline data, including sex, age, a history of hypertension and diabetes mellitus, smoking, alcohol consumption, and a pre-stroke mRS score, were collected through a face-to-face interview, which was conducted by an experienced neurologist. The baseline data for each participant were collected within 7 days of stroke onset.

The severity of the neurological deficit in each patient was evaluated by two appropriately trained neurologists who were not involved in this study using the National Institute of Health Stroke Scale (NIHSS) (22). The impairments of consciousness, gaze, visual field, facial palsy, arm and leg motor, limb ataxia, sensory, language, dysarthria, and level of neglect in each participant were scored, respectively. The sum of all sub-scores was the NIHSS score. When these neurologists disagreed on the scores, a third superior practitioner made the final assessment. The daily function of each patient was evaluated according to the mRS score (0–5 points) (23). During the hospitalization, all patients received medical treatments in accordance with the guidelines for the treatment of acute ischemic stroke (24). At discharge, the NIHSS and mRS scores of each patient were evaluated by a neurologist. A poor outcome refers to a mRS >2 points at discharge.

### 2.3. Evaluation of SSI by using cranial MRI

Each patient's infarct was evaluated using a 3.0T superconducting nuclear magnetic resonance apparatus (MAGNETOM Trio, Siemens, Germany). Imaging sequences consisted of T1-weighted imaging (T1WI), T2-weighted imaging (T2WI), fluid-attenuated inversion recovery (FLAIR), and diffusion-weighted imaging (DWI) (slice thickness: 0.8 mm; interval: 0 mm; field of view: 230 × 230 mm). The most common parameters used in the imaging sequences were as follows: T1WI: repetition time (TR) = 450 ms, echo time (TE) = 10 ms, total acquisition time: 1–2 min; T2WI: TR = 4,350 ms, TE = 95 ms, total acquisition time: 1–2 min; FLAIR:TR = 8,200 ms, TE = 113 ms, total acquisition time: 1–2 min; and DWI:TR = 4,000 ms, TE = 97 ms, total acquisition time: 1–2 min.

A single perforating infarct in the territory of the middle cerebral or basilar artery detected by diffusion-weighted imaging is defined as an SSI (1). The location of SSI in the territory of the middle cerebral artery is limited to the basal ganglia, corona radiata, or internal capsule (25), and the SSI in the territory of the basilar artery is limited to the paramedian pontine area (26). Because the lateral medullary infarct is generally associated with vertebral arterial dissection, the perforating infarct of the vertebral artery is limited to the medial medulla oblongata (27). However, patients with a medial medullary infarct are rare in clinical practice (28). Therefore, we excluded all patients with a medullary infarct from this study, which was in line with a previous study (1). The infarct size of the SSI of each participant was measured, which was represented by the largest lesional diameter on the section with the largest infarct in diffusion-weighted imaging, as previously reported (29).

**Figure 1:**
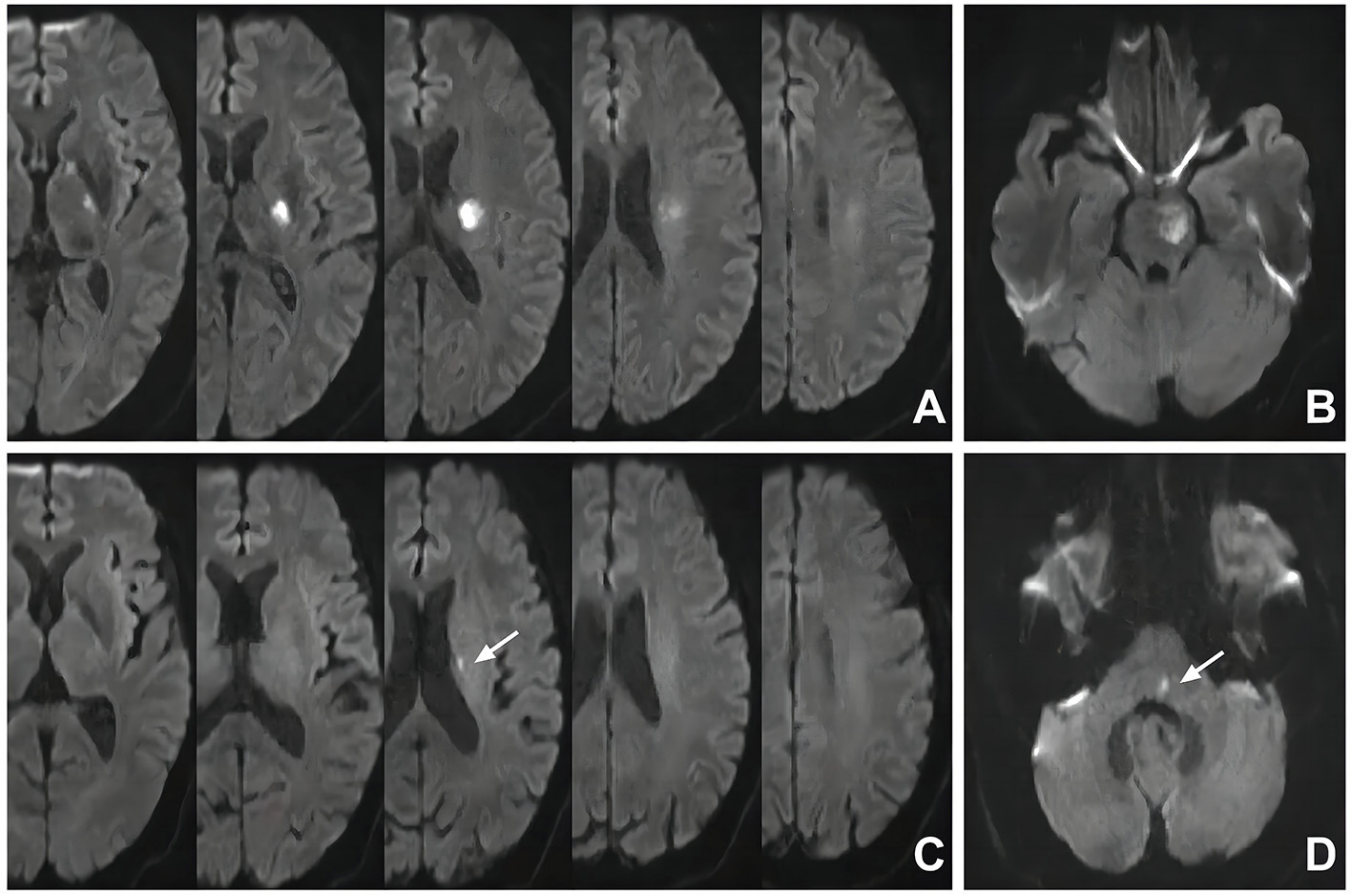
Representative cases of pSSI and dSSI. **(A)** pSSI in the territory of the middle cerebral artery: the SSI was involved with the basal ganglia; **(B)** pSSI in the territory of the basilar artery: the SSI was involved with the ventral part of the pons; **(C)** dSSI in the territory of the middle cerebral artery: the SSI was sited in the periventricular white matter without the involvement of the basal ganglia (white arrow); **(D)** dSSI in the territory of the basilar artery: the SSI was distal to the ventral part of the pons (white arrow). SSI, single subcortical infarction.

According to the positional relation between the SSI and the parental artery, SSIs can be dichotomized into proximal SSI (pSSI) and distal SSI (dSSI) (8, 9, 30). A pSSI is defined as an infarct located adjacent to the parental artery with an extension toward the basal surface of the parental artery, and a dSSI is defined as an infarct located only in the distal area of the parental artery (4, 31). For SSIs in the territory of the middle cerebral artery, involvement of the lowest portion of the basal ganglia is considered an extension to the basal surface of the parental artery; for SSIs in the territory of the basilar artery, involvement of the surface of the ventral pons is an extension to the basal surface of the parental artery (10, 32) (Figure 1).

### 2.4. Evaluation of PAD and aIAS by using MRA

The intracranial atherosclerotic conditions of all patients were evaluated through time-of-flight MRA. The most common parameters were as follows: flip angle = 20°; TR = 30 ms; TE = 10 ms; slice thickness = 1.2 mm; field of view = 230 mm; and total acquisition time = 3–5 min.

**Figure 2:**
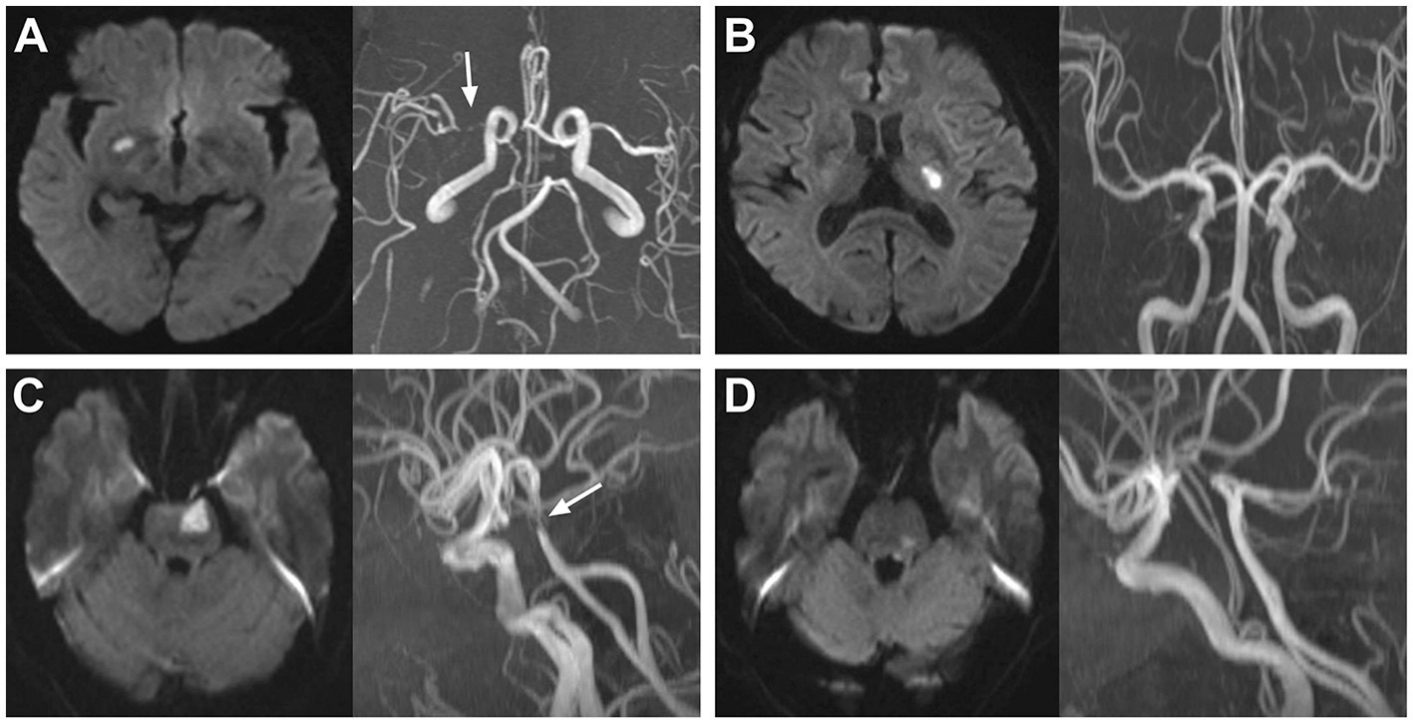
Representative cases of SSI patients with and without PAD. **(A)** SSI in the territory of the right middle cerebral artery with PAD (white arrow); **(B)** SSI in the territory of the left middle cerebral artery without PAD; **(C)** SSI in the territory of the basilar artery with PAD (white arrow); **(D)** SSI in the territory of the basilar artery without PAD. SSI, single subcortical infarction; PAD, parental arterial disease.

The intracranial large arteries under evaluation included the bilateral anterior cerebral (A_1_/A_2_ segments), middle cerebral (M_1_/M_2_ segments), posterior cerebral (P_1_/P_2_ segments), intracranial internal carotid, intracranial vertebral arteries, and the basilar artery. The degree of intracranial stenosis was evaluated according to the Warfarin-Aspirin Symptomatic Intracranial Disease Study Trial method (33). The formula for intracranial stenotic degree was as follows: stenotic degree = (Dn – Ds)/Dn × 100% (Ds: the luminal diameter of the narrowest site in the artery; Dn: the luminal diameter of the normal vessel proximal to the stenosis). The PAD is considered any degree of stenosis caused by atherosclerosis detected in the parental artery corresponding to the SSI (Figure 2) (8). When there was PAD, we considered it the symptomatic stenosis of SSI. An aIAS is defined as a stenosis of ≥50% (34) detected in an intracranial large artery without blood supply for the territory of SSI.

For SSIs in the territory of the middle cerebral artery, in the absence of PAD and the presence of a stenosis of ≥50% in the ipsilateral internal carotid artery, the SSI may be caused by this stenosis through artery-to-artery embolism; thus, this stenosis would not be defined as an aIAS. For SSIs in the territory of the basilar artery, in the absence of PAD but the presence of a stenosis of ≥50% in the ipsilateral vertebral artery, the SSI may be caused by this stenosis, which would not be defined as an aIAS, either. Two independent radiologists (Zhao T, who has work experience of 17 years, and Shao Y, who has work experience of 10 years) who were blinded to the clinical data of the patients assessed the intracranial atherosclerotic conditions. If there was disagreement between these two radiologists, a third superior practitioner would make the final diagnosis.

### 2.5. Statistical analysis

The sample size of the present study was determined using G^*^Power (version 3.1.9.7). This study aimed to compare the incidence of poor outcome between different groups, so we selected the chi-squared test as the statistical method and *a priori* as the power analysis. The input parameters were as follows: an effect size of 0.3, an α-error probability of 0.05, and a power of 0.95. The total sample size required was 220. Because the number of patients with SSI admitted into our stroke unit was approximately 300 per year, we decided to consecutively enroll the SSI patients admitted within 1 year into the present study.

The number of missing data was as follows: body mass index (7, 2.3%), triglyceride (3, 1.0%), total cholesterol (3, 1.0%), high-density lipoprotein cholesterol (3, 1.0%), low-density lipoprotein cholesterol (3, 1.0%), uric acid (4, 1.3%), glycosylated hemoglobin (7, 2.3%), homocysteine (14, 4.2%), neutrophil count (1, 0.3%), high-sensitivity C-reactive protein (13, 4.4%), and infarct size (2, 0.7%). Due to the relatively low rate of missing data, we considered the effect of it on the statistical results to be negligible. The missing data were interpolated with the predictive mean by using the expectation maximization (EM) method.

All statistical analyses were performed using SPSS version 25.0 (IBM, Armonk, NY, USA). The chi-squared test or Fisher's exact test was used for the comparison of the categorical variables. As described by the mean ± standard deviation, normally distributed continuous variables were compared using independent sample *t*-tests in two groups and a one-way analysis of variance test in multiple groups. As described by the median (interquartile range), non-normally distributed continuous variables were compared using Mann–Whitney *U* tests in two groups and Kruskal–Wallis tests in multiple groups.

After the univariable analyses, sex, age, and all factors with a *p*-value of < 0.05 were included in the modified Poisson regression model (35), and the correlations of clinical factors with the poor outcomes in patients with SSI and in the subgroups with and without PAD were analyzed. All tests were two-sided, and a *p*-value of < 0.05 was considered statistically significant.

## 3. Results

**Figure 3:**
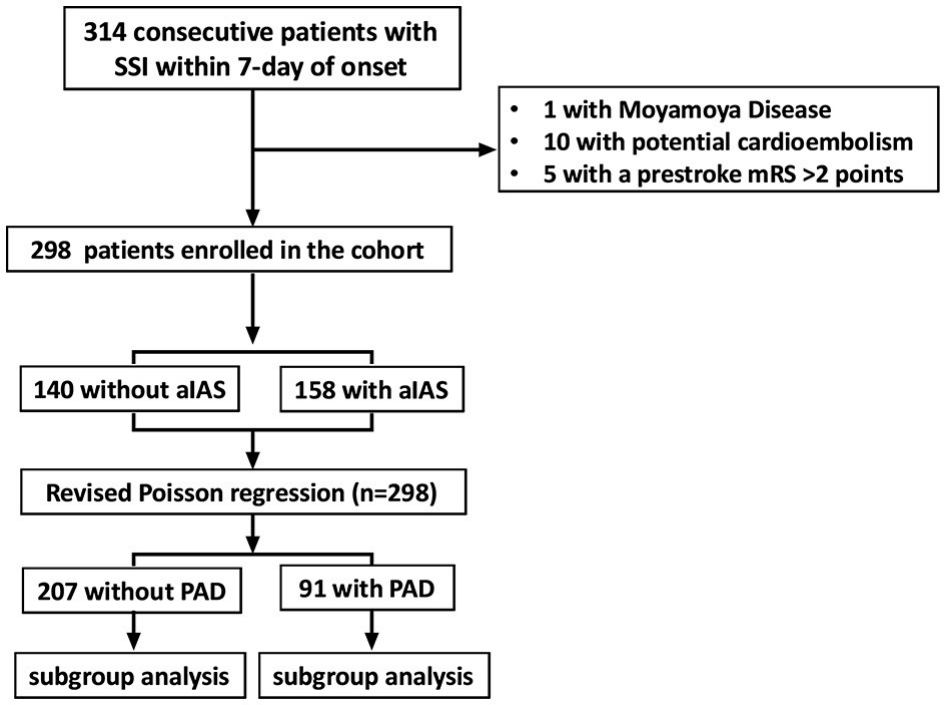
Flowchart of patients' enrollment. SSI, single subcortical infarction; mRS, modified Rankin Scale; aIAS, asymptomatic intracranial atherosclerotic stenosis; PAD, parental arterial disease.

The total sample size required in the present study was 220. Because the number of patients with SSI admitted to our stroke unit was approximately 300 per year, we decided to consecutively enroll the SSI patients admitted from 1 August 2021 to 31 August 2022 into the present study. A total of 314 patients with SSI within 7 days of onset were enrolled. Of these patients, one was diagnosed with Moyamoya disease, 10 with potential cardioembolism, and five with a prestroke mRS >2 points. These patients were excluded from this study. In total, 298 patients were finally enrolled in the cohort (Figure 3). The mean age was 65.6 ± 10.7 years, and 115 (38.6%) patients were women. The median of the initial NIHSS was 2.0 (1.0, 3.0) points. Cohen's kappa was used to determine the intraobserver and interobserver agreements in the evaluation of the NIHSS score. The κ values of the intraobserver and interobserver agreements were 1.00 and 0.816, respectively (both *p* < 0.001). Among these patients, 140 (47.0%) were without aIAS, 158 (53.0%) had aIAS, 91 (30.5%) had PAD, and 201 (69.5%) were without PAD. We also used Cohen's kappa test to examine the intraobserver and interobserver reliabilities in the evaluation of PAD and aIAS. The κ values of the intraobserver and interobserver agreements were 1.00 and 0.848 in the evaluation of PAD and were 1.00 and 0.794 in the evaluation of aIAS (all *p*-values < 0.001). At discharge, 234 (78.5%) patients had a good outcome (mRS ≤ 2 points), and 64 (21.5%) patients had a poor outcome (mRS > 2 points) (Table 1).

**Table 1:** Baseline characteristics of all participants.

**Baseline characteristics**	**Total (*n =* 298)**
Female, *n* (%)	115 (38.6)
Age (years), mean ± SD	65.6 ± 10.7
Hypertension, *n* (%)	206 (69.1)
Diabetes mellitus, *n* (%)	103 (34.6)
Smoking, *n* (%)	121 (40.6)
Alcohol consumption, *n* (%)	99 (33.2)
Prestroke mRS (points), median (IQR)	0 (0, 0)
BMI, mean ± SD	25.08 ± 3.38
SBP (mmHg), median (IQR)	153.0 (142.0, 169.0)
DBP (mmHg), median (IQR)	84.0 (75.0, 93.3)
TG (mmol/L), median (IQR)	1.44 (1.07, 2.00)
TC (mmol/L), mean ± SD	4.64 ± 1.02
HDL-C (mmol/L), median (IQR)	1.03 (0.85, 1.28)
LDL-C (mmol/L), mean ± SD	2.72 ± 0.86
Uric acid (mmol/L), median (IQR)	295.60 (242.93, 381.13)
HbA1c (%), median (IQR)	6.20 (5.80, 7.90)
Homocysteine (mmol/L), median (IQR)	11.39 (9.27, 14.57)
Neutrophil count (× 10^9^/L), median (IQR)	4.30 (3.45, 5.50)
Hs-CRP (mg/L), median (IQR)	1.00 (0.50, 2.40)
Initial NIHSS (point), median (IQR)	2.0 (1.0, 3.0)
Intravenous thrombolysis, *n* (%)	2 (0.7)
Presence of PAD, *n* (%)	91 (30.5)
Presence of aIAS, *n* (%)	158 (53.0)
pSSI, *n* (%)	209 (70.1)
Infarct size (mm), median (IQR)	13.83 (10.20, 19.25)
Anti-platelet, *n* (%)	282 (94.6)
Statins, *n* (%)	295 (99.0)
Posterior lesion, *n* (%)	97 (32.6)
Hospital stay (day), median (IQR)	10.0 (8.0, 13.0)
Poor outcome, *n* (%)	64 (21.5%)

aIAS, asymptomatic intracranial atherosclerotic stenosis; mRS, modified Ranking Scale; BMI, body mass index; SBP, systolic blood pressure; DBP, diastolic blood pressure; TG, triglyceride; TC, total cholesterol; HDL-C, high-density lipoprotein cholesterol; LDL-C, low-density lipoprotein cholesterol; HbA1c, glycosylated hemoglobin; hs-CRP, high-sensitivity C-reactive protein; NIHSS, National Institutes of Health Stroke Scale; PAD, parental arterial disease; pSSI, proximal single subcortical infarction.

### 3.1. Comparisons of clinical characteristics in patients with and without aIAS

**Figure 4:**
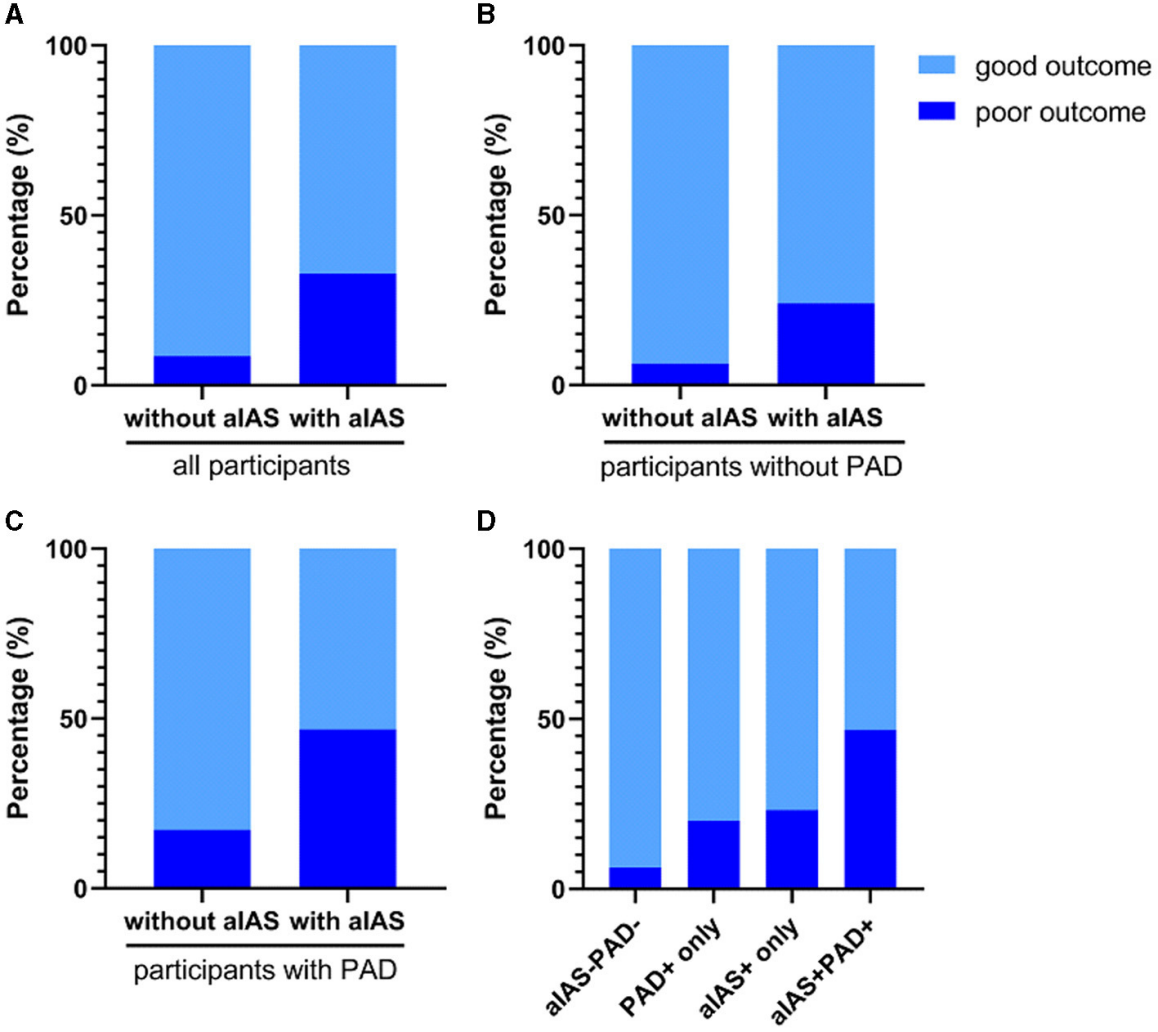
Comparisons of poor outcome in different groups. **(A)** The proportion of poor outcome was higher in the group with aIAS than without aIAS (32.9 vs. 8.6%, *p* < 0.001); **(B)** patients with aIAS had a higher proportion of poor outcome than those without aIAS in the subgroup without PAD (24.0 vs. 6.3%, *p* < 0.001); **(C)** the proportion of poor outcome in patients with aIAS was significantly higher than those without aIAS in the subgroup with PAD (46.8 vs. 17.2%, *p* = 0.007); **(D)** the proportion of poor outcome was significantly different among the groups divided according to the presence of PAD and/or aIAS (46.8 vs. 6.3% vs. 20.0 vs. 23.2%, *p* < 0.001). All *p*-values were calculated using the chi-squared test. aIAS, asymptomatic intracranial atherosclerotic stenosis; PAD, parental arterial disease.

All patients were classified into groups without (*n* = 140) and with (*n* = 158) aIAS. In the univariable analyses, the age (62.9 ± 10.3 vs. 68.0 ± 10.5 years), the proportions of female patients (28.6 vs. 47.5%), a history of hypertension (57.1 vs. 79.7%) and diabetes mellitus (43.0 vs. 25.0%), smoking (50.7 vs. 31.6%) and alcohol consumption (40.7 vs. 26.6%), the prestroke mRS [0.0 (0.0, 0.0) vs. 0.0 (0.0, 0.0) points], diastolic blood pressure at admission [85.0 (76.3, 94.0) vs. 82.0 (74.0, 91.0) mmHg], the levels of high-density lipoprotein cholesterol [0.97 (0.81, 1.18) vs. 1.06 (0.89, 1.35) mmol/L], and glycosylated hemoglobin [6.10 (5.80, 7.60) vs. 6.55 (5.80, 8.35) %], initial NIHSS scores [2.0 (1.0, 3.0) vs. 2.0 (1.0, 4.0) points], the proportion of PAD (20.7% vs. 39.2%), infarct size [11.93 (9.43, 17.38) vs. 15.41 (10.62, 21.84) mm], the proportion of posterior SSI (23.6% vs. 40.5%), and hospital stay [10.0 (8.0, 12.0) vs. 11.0 (9.0, 13.0) days] showed significant difference between these two groups (all *p*s < 0.05) (Table 2). Compared with the group without aIAS, the proportion of poor outcome increased in the group with aIAS (32.9% vs. 8.6%, *p* < 0.001; Figure 4A).

**Table 2:** Comparisons of clinical characteristics between patients without and with aIAS.

**Clinical characteristics**	**Without aIAS (*n =* 140)**	**With aIAS (*n =* 158)**	***P*-value**
Female, *n* (%)	40 (28.6)	75 (47.5)	0.001^*^
Age (years), mean ± SD	62.9 ± 10.3	68.0 ± 10.5	< 0.001^*^
Hypertension, *n* (%)	80 (57.1)	126 (79.7)	< 0.001^*^
Diabetes mellitus, *n* (%)	35 (25.0)	68 (43.0)	0.001^*^
Smoking, *n* (%)	71 (50.7)	50 (31.6)	0.001^*^
Alcohol consumption, *n* (%)	57 (40.7)	42 (26.6)	0.01^*^
Prestroke mRS (points), median (IQR)	0 (0, 0)	0 (0, 0)	0.047^*^
BMI, mean ± SD	25.35 ± 3.15	24.84 ± 3.56	0.20
SBP (mmHg), median (IQR)	152.5 (140.3, 166.8)	154.0 (145.0, 171.0)	0.22
DBP (mmHg), median (IQR)	85.0 (76.3, 94.0)	82.0 (74.0, 91.0)	0.027^*^
TG (mmol/L), median (IQR)	1.44 (1.01, 2.13)	1.44 (1.13, 1.92)	0.97
TC (mmol/L), mean ± SD	4.67 ± 0.98	4.62 ± 1.07	0.71
HDL-C (mmol/L), median (IQR)	0.97 (0.81, 1.18)	1.06 (0.89, 1.35)	0.005^*^
LDL-C (mmol/L), mean ± SD	2.74 ± 0.85	2.71 ± 0.88	0.81
Uric acid (mmol/L), median (IQR)	296.20 (252.60, 387.0)	294.00 (235.00, 377.20)	0.65
HbA1c (%), median (IQR)	6.10 (5.80, 7.60)	6.55 (5.80, 8.35)	0.032^*^
Homocysteine (mmol/L), median (IQR)	11.75 (9.45, 14.59)	11.09 (8.94, 14.79)	0.52
Neutrophil count (× 10^9^/L), median (IQR)	4.30 (3.40, 5.20)	4.40 (3.65, 5.70)	0.13
Hs-CRP (mg/L), median (IQR)	0.90 (0.50, 2.20)	1.10 (0.50, 2.93)	0.50
Initial NIHSS (point), median (IQR)	2.0 (1.0, 3.0)	2.0 (1.0, 4.0)	0.006^*^
Intravenous thrombolysis, *n* (%)	0 (0.0)	2 (1.3)	0.53
Presence of PAD, *n* (%)	29 (20.7)	62 (39.2)	0.001^*^
pSSI, *n* (%)	92 (65.7)	117 (74.1)	0.12
Infarct size (mm), median (IQR)	11.93 (9.43, 17.38)	15.41 (10.62, 21.84)	< 0.001^*^
Anti-platelet, *n* (%)	135 (96.4)	147 (93.0)	0.20
Statins, *n* (%)	139 (99.3)	156 (98.7)	1.00
Posterior lesion, *n* (%)	33 (23.6)	64 (40.5)	0.002^*^
Hospital stay (day), median (IQR)	10.0 (8.0, 12.0)	11.0 (9.0, 13.0)	0.02^*^

aIAS, asymptomatic intracranial atherosclerotic stenosis; mRS, modified Ranking Scale; BMI, body mass index; SBP, systolic blood pressure; DBP, diastolic blood pressure; TG, triglyceride; TC, total cholesterol; HDL-C, high-density lipoprotein cholesterol; LDL-C, low-density lipoprotein cholesterol; HbA1c, glycosylated hemoglobin; hs-CRP, high-sensitivity C-reactive protein; NIHSS, National Institutes of Health Stroke Scale; PAD, parental arterial disease; pSSI, proximal single subcortical infarction. ^*^p < 0.05 was considered statistically significant.

### 3.2. The association of aIAS with the poor outcome of all SSI patients

In the modified Poisson regression analysis, after adjusting for aforementioned confounders, the presence of aIAS [adjusted relative risk (aRR) = 2.14, 95% confidence interval (CI) = 1.17–3.93, *p* = 0.014] could independently predict the poor outcome of all SSI patients. Other predictors of the poor outcome included the initial NIHSS (aRR = 1.20, 95% CI = 1.15–1.25, *p* < 0.001), infarct size (aRR = 1.04, 95% CI = 1.02–1.06, *p* < 0.001), and hospital stay (aRR = 1.09, 95% CI = 1.04–1.14, *p* < 0.001) (Table 3).

**Table 3:** Independent predictors of the poor outcome of patients with SSI.

**Predictors**	**Poor outcome of SSI patients**			
	**Crude RR (95%CI)**	***P*-value**	**Adjusted RR^†^ (95%CI)**	***P*-value**
Presence of aIAS	3.65 (2.03–6.57)	< 0.001^*^	2.14 (1.17–3.93)	0.014^*^
Initial NIHSS	1.26 (1.20–1.32)	< 0.001^*^	1.20 (1.15–1.25)	< 0.001^*^
Infarct size	1.06 (1.04–1.08)	< 0.001^*^	1.04 (1.02–1.06)	< 0.001^*^
Hospital stay	1.15 (1.11–1.20)	< 0.001^*^	1.09 (1.04–1.14)	< 0.001^*^

^†^Sex, age, a history of hypertension and diabetes mellitus, smoking, alcohol consumption, pre-stroke mRS score, diastolic blood pressure at admission, the levels of high-density lipoprotein cholesterol and glycosylated hemoglobin, initial NIHSS, infarct size, posterior SSI, hospital stay, presence of PAD and aIAS were simultaneously included in the model. SSI, single subcortical infarction; aIAS, asymptomatic intracranial atherosclerotic stenosis; NIHSS, National Institutes of Health Stroke Scale. ^*^p < 0.05 was considered statistically significant.

### 3.3. The association of aIAS with the poor outcome in SSI patients without PAD

Of all 298 patients with SSI, 207 (69.5%) were without PAD, and these patients were classified into groups without (*n* = 111) and with aIAS (*n* = 96). In this subgroup without PAD, patients with aIAS had a higher proportion of poor outcome than those without aIAS (24.0 vs. 6.3%, *p* < 0.001, Figure 4B). In univariable analyses, age, the proportion of female patients, a history of hypertension and diabetes mellitus, smoking, diastolic blood pressure at admission, infarct size, and the proportion of posterior SSI were significantly different between these two groups (Supplementary Table 1). In modified Poisson regression analysis, after adjusting for the aforementioned factors, the presence of aIAS (aRR = 3.12, 95% CI = 1.47–6.62, *p* = 0.003) and infarct size (aRR = 1.12, 95% CI = 1.08–1.16, *p* < 0.001) could independently predict the poor outcome of SSI patients without PAD (Table 4).

**Table 4:** Independent predictors of the poor outcome of SSI patients without and with PAD.

**Clinical factors**	**Poor outcome**			
	**Without PAD** ^**†**^		**With PAD** ^**‡**^	
	**Adjusted^†^ RR (95%CI)**	***P*-value**	**Adjusted^‡^ RR (95%CI)**	***P*-value**
Presence of aIAS	3.12 (1.47–6.62)	0.003^*^	1.28 (0.57–2.89)	0.56
Female	–		2.03 (1.04–3.95)	0.037^*^
Initial NIHSS	–		1.18 (1.11–1.25)	< 0.001^*^
Infarct size	1.12 (1.08–1.16)	< 0.001^*^	1.04 (1.01–1.06)	0.007^*^

^†^Age, sex, a history of hypertension and diabetes mellitus, smoking, diastolic blood pressure at admission, infarct size, posterior SSI and the presence of aIAS were simultaneously included in the model. ^‡^Age, sex, smoking, the level of high-density lipoprotein cholesterol, initial NIHSS, infarct size, posterior SSI, and the presence of aIAS were simultaneously included in the model. SSI, single subcortical infarction; PAD, parental arterial disease; aIAS, asymptomatic intracranial atherosclerotic stenosis; NIHSS, National Institutes of Health Stroke Scale. ^*^p < 0.05 was considered statistically significant.

### 3.4. The association of aIAS with the poor outcome in SSI patients with PAD

Of all 298 patients with SSI, 91 (30.5%) had PAD, and these patients were also classified into groups without (*n* = 29) and with aIAS (*n* = 62). The proportion of poor outcome in the group with aIAS was significantly higher than in the group without aIAS (46.8 vs. 17.2%, *p* = 0.007; Figure 4C). After adjusting for sex and variables that showed significant differences in univariable analyses (including age, the proportion of smoking, the level of high-density lipoprotein cholesterol, initial NIHSS, infarct size, and the proportion of posterior SSI; Supplementary Table 2), the presence of aIAS (aRR = 1.28, 95% CI = 0.57–2.89, *p* = 0.56) was not independently correlated with the poor outcome of SSI patients with PAD (Table 4). In this subgroup, the predictors of the poor outcome were female patients (aRR = 2.03, 95% CI = 1.04–3.95, *p* = 0.037), initial NIHSS (aRR = 1.18, 95% CI = 1.11–1.25, *p* < 0.001), and infarct size (aRR = 1.04, 95% CI = 1.01–1.06, *p* = 0.007) (Table 4).

### 3.5. The association of concomitant aIAS and PAD with the poor outcome in patients with SSI

All 298 patients were divided into four groups according to the presence of aIAS and/or PAD: group with neither aIAS nor PAD (aIAS^−^PAD^−^, *n* = 111), group with PAD only (PAD^+^ only, *n* = 30), group with aIAS only (aIAS^+^ only, *n* = 95), and group with concomitant aIAS and PAD (aIAS^+^PAD^+^, *n* = 62). Compared with the other three groups, the proportion of patients with a poor outcome was significantly higher in the aIAS^+^PAD^+^ group (46.8 vs. 6.3% vs. 20.0 vs. 23.2%, *p* < 0.001; Figure 4D). Among the four groups, age, sex, the proportions of a history of hypertension and diabetes mellitus, smoking and alcohol consumption, the level of high-density lipoprotein cholesterol, the initial NIHSS, hospital stay, and the proportion of posterior infarct showed significant differences (Supplementary Table 3).

When all aforementioned factors were adjusted in multivariate logistic regression analysis, compared with the aIAS^−^PAD^−^ group, the risk of a poor outcome increased approximately 2-fold in the PAD^+^ only group (aRR = 3.56, 95% CI = 1.46–8.67, *p* = 0.005), in the aIAS^+^ only group (aRR = 2.95, 95% CI = 1.55–5.60, *p* = 0.001), and in the aIAS^+^PAD^+^ group (aRR = 3.10, 95% CI = 1.62–5.95, *p* = 0.001) (Table 5).

**Table 5:** The predictive value of the presence of aIAS and/or PAD for poor outcome of SSI patients.

**Clinical factors**	**Poor outcome of SSI patients**			
	**Crude RR (95%CI)**	***P*-value**	**Adjusted RR^†^ (95%CI)**	**P-value**
Neither aIAS nor PAD	Ref.	–	Ref.	–
PAD only	3.25 (1.18–8.93)	0.022^*^	3.56 (1.46–8.67)	0.005^*^
aIAS only	3.72 (1.66–8.31)	0.001^*^	2.95 (1.55–5.60)	0.001^*^
Concomitant aIAS and PAD	7.21 (3.35–15.53)	< 0.001^*^	3.10 (1.62–5.95)	0.001^*^

^†^Adjusted for age, sex, hypertension and diabetes mellitus history, smoking, alcohol consumption, HDL-C, initial NIHSS, posterior stroke, and hospital stay. aIAS, asymptomatic intracranial atherosclerotic stenosis; SSI, single subcortical infarction; PAD, parental arterial disease. ^*^p < 0.05 was considered statistically significant.

## 4. Discussion

In this study, we included the presence of aIAS and PAD and confounders that might affect the outcome of SSI patients in modified Poisson regression models to explore the predictive value of the presence of aIAS for the post-stroke disability of patients with SSI and those without PAD. After adjusting for all confounders, we found the presence of aIAS to be an independent predictor of post-stroke disability in patients with SSI. In the subgroup analysis, the presence of aIAS could predict a poor outcome in SSI patients without PAD but failed in those with PAD. Compared with SSI patients with neither PAD nor aIAS, those with sole PAD, sole aIAS, or concomitant aIAS and PAD had an approximately 2-fold increased risk of a poor outcome.

Previous studies focused on the effect of aIAS on the clinical outcomes of patients with SSI are rare. Han reported that the presence of aIAS is associated with the occurrence of early ND in patients with SSI (10). However, this study did not investigate the relationship between the presence of aIAS and the poor outcomes of its study subjects. In addition, this study only enrolled patients with posterior SSI. In the present study, we enrolled patients with either anterior or posterior SSI and demonstrated that the presence of aIAS is an independent predictor of the poor outcomes of patients with SSI. SSI patients with aIAS had a larger infarct size than those without aIAS, and a large infarct lesion was also demonstrated as an independent predictor of the poor outcome of patients with SSI in the present study. This could interpret the relationship between the presence of aIAS and the poor outcomes of patients with SSI. In addition, as we mentioned above, previous work corroborated that intracranial significant stenosis could compromise the perfusion in the predilection sites of SSI, including the basal ganglia and centrum ovale (18). The compromised perfusion caused by significant stenosis in these sites may further impair the neurons in the ischemic tissues and ultimately result in the poor outcomes of patients with SSI. This is a potential reason for the detrimental effect of aIAS on the clinical outcomes of patients with SSI.

We demonstrated the presence of PAD was predictive of the poor outcomes of patients with SSI in a previous study (2). However, in the present study, the presence of PAD was not an independent predictor of the poor outcome of SSI patients after adjusting for the presence of aIAS, infarct size, and other confounders in the multivariable analysis. This disagreement between these two studies is likely to be attributed to the different study subjects, study designs, and statistical methods. Given that PAD is closely correlated with infarct formation and that the pathological progress of PAD could significantly affect the outcomes in patients with SSI (8), we still considered the presence of PAD as a key factor associated with the outcomes of these patients. The incidence of PAD accounts for 30.5% in the present study, which is in line with previous work (9). As we mentioned above, due to a low incidence of PAD in patients with SSI, the factors that can predict the clinical outcomes of SSI patients without PAD seem to be limited. Hence, we further explored whether the presence of aIAS could independently predict the clinical outcomes in those SSI patients without PAD. We classified all participants into groups with and without PAD and found that the presence of aIAS could predict the poor outcome in participants without PAD but not in those with PAD. For SSI patients without PAD, the predilection site of the SSI was the periventricular white matter, i.e., the deep white matter. Previous work reported that the presence of intracranial significant stenosis of ≥50% was correlated with the severity of deep white matter hyperintensities, which denoted insufficient perfusion for the deep white matter (36). This suggested the presence of aIAS might impair the axons of motor neurons, which hampered the functional recovery of SSI patients without PAD. For SSI patients with PAD, the presence of aIAS is, however, disassociated with clinical outcomes. The plausible reason is that the clinical outcomes of these patients are likely to be associated with the characteristics and pathological progress of the atherosclerotic plaque(s) in the parental artery (6, 37) and are consequently irrelevant to the presence of aIAS.

Overall, we demonstrated in the present study that the presence of aIAS was an independent predictor of post-stroke disability in all SSI populations, whereas the presence of PAD was not. In the subgroup analyses, the presence of aIAS could predict the post-stroke disability of SSI patients without PAD, but failed in those with PAD. Finally, we divided all participants into four groups according to the presence of PAD and/or aIAS. Compared with SSI patients with neither PAD nor aIAS, patients with sole PAD, sole aIAS, or concomitant aIAS and PAD have an approximately 2-fold increased risk of post-stroke disability. This indicates that a risk stratification of post-stroke disability based on the presence of aIAS is feasible for patients with SSI, especially for those without PAD. For SSI patients without PAD, the evaluation of aIAS is essential in clinical practice, which could contribute to the early identification of those with a high risk of post-stroke disability. For these patients, intensive management of the intracranial significant stenosis caused by atherosclerosis, e.g., high-intensity statins, the proprotein convertase subtilisin/kexin 9 (PCSK9) inhibitor, or even endovascular therapy, may be beneficial for the post-stroke functional recovery. A further randomized clinical trial to explore the effect of these therapies on the outcomes of SSI patients with aIAS would be important.

Some scholars have suggested dividing the SSIs into pSSI and dSSI according to the positional correlation between the infarct and the parental artery (9). A previous study reported that the risk of post-stroke disability in patients with pSSI is higher than in patients with dSSI (30). However, pSSI is irrelevant to the poor outcomes in all patients with SSI or in the subgroup with or without PAD in the present study, which is inconsistent with the previous study. This divergence may be due to the different inclusion criteria between the present and previous studies. The previous study only enrolled patients with SSI located in the territory of the middle cerebral artery (30), whereas the present study enrolled patients with either anterior or posterior SSI in the cohort.

In the present study, the proportion of female patients in the group with concomitant aIAS and PAD is higher than in the other three groups, and this denotes that female patients with SSI are more likely to suffer from intracranial atherosclerosis than male patients. This result is in accordance with a previous study (38) and is caused by differences in hormone levels and vessel sizes between the two sexes (39). Interestingly, we found the proportions of smoking and alcohol consumption were lower in the concomitant aIAS and PAD group than in the other three groups, and this result is likely to be associated with the higher proportion of female patients in this group.

The present study has some strengths. The proportion of SSI patients without PAD is relatively high in the whole SSI population, but the predictors of clinical outcomes are limited in these patients. This study demonstrated that the presence of aIAS could independently predict the post-stroke disability of SSI patients without PAD. The results contribute to the early identification of those SSI patients without PAD but with a potentially high risk of post-stroke disability. Furthermore, the results of the present study could to some extent enlighten the neurologists in the administrations for SSI patients without PAD. For these SSI patients without PAD, an intensive management for intracranial signiicant stenosis may be beneicial. There are several limitations to this study. First, this is a single-center study, and the majority of the participants belong to the Chinese Han population. The results of the present study may not be applicable to other ethnic groups or regions, and the generalization of the results of this study needs to be cautious. Second, the sample sizes of some subgroups in this study, especially the subgroup with PAD only, are relatively small. A further study with a larger sample size would be conducted to validate the predictive value of the presence of aIAS on the outcomes of patients with SSI. Third, the presence of PAD is determined through the evaluation of the stenotic degree of the parental artery, which is identified by using time-of-flight MRA. However, a large artery with a normal luminal diameter in MRA may also have an eccentric plaque (6), so the present study may underestimate the incidence of PAD. A subsequent study needs to evaluate the PAD by using high-resolution MRI to accurately explore the associations between the presence of PAD and aIAS and the clinical outcomes of patients with SSI. In addition, the time-of-flight MRA is prone to artifacts caused by blood flow abnormalities, and the assessment of stenosis may be hampered by blood flow velocity. However, this method is still widely used in clinical research because of its non-invasiveness and accessibility. Finally, the infarct size is represented by the largest diameter of the SSI in the present study, instead of being calculated by using specific software. A previous study has corroborated that the largest ischemic lesional diameter could relatively relect the infarct volume (40), but a a further study with accurate calculations of the infarct volumes is still needed, for more precisely exploring the association between the presence of aIAS and the outcomes of patients with SSI.

## 5. Conclusion

The presence of aIAS could be considered a predictor of poststroke disability in patients with SSI, especially in those without PAD. The evaluation of aIAS contributes to providing a potential risk stratification of poststroke disability for patients with SSI and to developing personalized administrations for SSI patients with different risks of post-stroke disability. Further studies with a larger sample size, a more advanced neuroimaging method to evaluate the presence of aIAS, e.g., high-resolution MRI, and a long-term follow-up would be conducted to validate the predictive value of aIAS for the outcomes of patients with SSI.

## Data availability statement

The original contributions presented in the study are included in the article/Supplementary material, further inquiries can be directed to the corresponding author.

## Ethics statement

The studies involving humans were approved by the Research Ethics Committee of Affiliated Hospital of Jiangsu University. The studies were conducted in accordance with the local legislation and institutional requirements. The participants provided their written informed consent to participate in this study.

## Author contributions

YY and YH: design and writing. YX and WH: visualization. TZ and YS: methods and resources. YY: revising. MY: design, revising, reviewing, supervision, and editing of the final version of the manuscript. All authors contributed to the article and approved the submitted version.
