# Supplementary material for: The impact of asymptomatic intracranial atherosclerotic stenosis on the clinical outcomes of patients with single subcortical infarction
Source: Front Med (Lausanne). 2023 Sep 1;10:1249347. doi: 10.3389/fmed.2023.1249347 (PMC10502720; doi:10.3389/fmed.2023.1249347)
Supplement: Supplementary file 2 [file Table_1.DOCX]

Supplementary Material

**The impact of asymptomatic intracranial atherosclerotic stenosis on the clinical outcomes of patients with single subcortical infarction**

Yi Yang^†^, Yue He^†^, Yuhao Xu, Wei Han, Yuanwei Shao, Tian Zhao, Ming Yu^*^

† These authors contributed equally to this work and share first authorship.

*** Correspondence:** Ming Yu: yuming7251@163.com

# Supplementary Tables

Supplementary Table S1. Comparisons of clinical characteristics between patients without and with aIAS in the subgroup without PAD

| Clinical characteristics | Patients without PAD (n=207) | | |
| --- | --- | --- | --- |
|  | Without aIAS (n=111) | With aIAS (n=96) | P-value |
| Female, n (%) | 28 (25.2) | 37 (38.5) | 0.04 |
| Age (year), mean ± SD | 62.7±10.8 | 67.2±10.4 | 0.003 |
| Hypertension, n (%) | 62 (55.9) | 77 (80.2) | < 0.001 |
| Diabetes mellitus, n (%) | 27 (24.3) | 40 (41.7) | 0.008 |
| Smoking, n (%) | 57 (51.4) | 36 (37.5) | 0.046 |
| Alcohol consumption, n (%) | 48 (43.2) | 31 (32.3) | 0.11 |
| Pre-stroke mRS (points), median (IQR) | 0 (0, 0) | 0 (0, 0) | 0.066 |
| BMI, median (IQR) | 25.39 (23.16, 27.34) | 24.79 (22.63, 27.34) | 0.27 |
| SBP (mmHg), median (IQR) | 152.0 (140.0, 167.0) | 150.5 (142.0, 162.0) | 0.98 |
| DBP (mmHg), median (IQR) | 87.0 (78.0, 95.0) | 81.0 (74.0, 92.0) | 0.031 |
| TG (mmol/L), median (IQR) | 1.42 (1.02, 2.09) | 1.46 (1.12, 2.03) | 0.79 |
| TC (mmol/L), mean ± SD | 4.66±0.96 | 4.54±1.01 | 0.38 |
| HDL-C (mmol/L), median (IQR) | 0.97 (0.83, 1.19) | 1.05 (0.86, 1.33) | 0.11 |
| LDL-C (mmol/L), mean ± SD | 2.73±0.87 | 2.66±0.83 | 0.58 |
| Uric acid (mmol/L), median (IQR) | 294.00  (253.00, 371.00) | 294.00  (241.95, 359.45) | 0.83 |
| HbA1c (%), median (IQR) | 6.10 (5.80, 7.50) | 6.40 (5.80, 8.40) | 0.052 |
| Homocysteine (mmol/L), median (IQR) | 11.45 (9.28, 14.21) | 10.95 (9.06, 14.57) | 0.63 |
| Neutrophil count (×10^9^/L), median (IQR) | 4.30 (3.40, 5.40) | 4.30 (3.50, 5.70) | 0.66 |
| Hs-CRP (mg/L), median (IQR) | 0.80 (0.50, 2.20) | 0.90 (0.50, 2.30) | 0.96 |
| Initial NIHSS (point), median (IQR) | 2.0 (1.0, 3.0) | 2.0 (1.0, 3.0) | 0.32 |
| pSSI, n (%) | 72 (64.9) | 66 (68.8) | 0.55 |
| Intravenous thrombolysis, n (%) | 0 (0.0) | 2 (2.1) | 0.42 |
| Infarct size (mm), median (IQR) | 11.80 (9.74, 16.82) | 14.49 (10.64, 19.30) | 0.014 |
| Anti-platelet, n (%) | 107 (96.4) | 90 (93.8) | 0.58 |
| Statins, n (%) | 110 (99.1) | 96 (100.0) | 1.00 |
| Posterior lesion, n (%) | 29 (26.1) | 43 (44.8) | 0.005 |
| Hospital stay (day), median (IQR) | 10.0 (8.0, 12.0) | 10.0 (9.0, 12.0) | 0.19 |

Abbreviations: aIAS indicates asymptomatic intracranial atherosclerotic stenosis; PAD, parental arterial disease; mRS, modified Ranking Scale; BMI, body mass index; SBP, systolic blood pressure; DBP, diastolic blood pressure; TG, triglyceride; TC, total cholesterol; HDL-C, high-density lipoprotein cholesterol; LDL-C, low-density lipoprotein cholesterol; HbA1c, glycosylated hemoglobin; hs-CRP, high-sensitivity C-reactive protein; NIHSS, National Institutes of Health Stroke Scale; pSSI, proximal single subcortical infarction.

^*^ *p* < 0.05 was considered statistically significant.

Supplementary Table S2. Comparisons of clinical characteristics between patients without and with aIAS in the subgroup with PAD

| Clinical characteristics | Patients with PAD (n=91) | | |
| --- | --- | --- | --- |
|  | Without aIAS (n=29) | With aIAS (n=62) | P-value |
| Female, n (%) | 12 (41.4) | 38 (61.3) | 0.075 |
| Age (year), mean ± SD | 63.5±8.6 | 69.2±10.7 | 0.014 |
| Hypertension, n (%) | 18 (62.1) | 49 (79.0) | 0.087 |
| Diabetes mellitus, n (%) | 8 (27.6) | 28 (45.2) | 0.11 |
| Smoking, n (%) | 14 (48.3) | 14 (22.6) | 0.013 |
| Alcohol consumption, n (%) | 9 (31.0) | 11 (17.7) | 0.15 |
| Pre-stroke mRS (points), median (IQR) | 0 (0, 0) | 0 (0, 0) | 0.33 |
| BMI, median (IQR) | 24.98 (22.53, 27.23) | 24.22 (22.23, 26.62) | 0.60 |
| SBP (mmHg), median (IQR) | 153.0 (144.0, 165.5) | 162.0 (147.0, 174.0) | 0.076 |
| DBP (mmHg), median (IQR) | 83.0 (74.0, 89.0) | 82.5 (73.3, 91.0) | 0.82 |
| TG (mmol/L), median (IQR) | 1.55 (0.99, 2.39) | 1.42 (1.13, 1.84) | 0.57 |
| TC (mmol/L), mean ± SD | 4.69±1.05 | 4.75±1.14 | 0.81 |
| HDL-C (mmol/L), median (IQR) | 0.94 (0.80, 1.12) | 1.18 (0.93, 1.40) | 0.021 |
| LDL-C (mmol/L), mean ± SD | 2.78±0.80 | 2.80±0.94 | 0.96 |
| Uric acid (mmol/L), median (IQR) | 332.00  (240.88, 425.53) | 296.50  (233.73, 401.25) | 0.38 |
| HbA1c (%), median (IQR) | 6.20 (5.85, 8.50) | 6.80 (5.80, 8.45) | 0.51 |
| Homocysteine (mmol/L), median (IQR) | 12.28 (9.52, 15.10) | 11.22 (8.93, 15.31) | 0.64 |
| Neutrophil count (×10^9^/L), median (IQR) | 4.20 (3.35, 5.00) | 4.60 (3.78, 5.88) | 0.054 |
| Hs-CRP (mg/L), median (IQR) | 1.10 (0.60, 1.70) | 1.50 (0.50, 3.60) | 0.28 |
| Initial NIHSS (point), median (IQR) | 1.0 (0.0, 3.0) | 3.0 (1.0, 5.0) | 0.004 |
| pSSI, n (%) | 20 (69.0) | 51 (82.3) | 0.15 |
| Intravenous thrombolysis, n (%) | 0 | 0 | – |
| Infarct size (mm), median (IQR) | 14.72 (8.61, 19.01) | 17.26 (10.36, 25.64) | 0.032 |
| Anti-platelet, n (%) | 28 (96.6) | 57 (91.9) | 0.71 |
| Statins, n (%) | 29 (100.0) | 60 (96.8) | 0.83 |
| Posterior lesion, n (%) | 4 (13.8) | 21 (33.9) | 0.046 |
| Hospital stay (day), median (IQR) | 11.0 (9.0, 13.0) | 12.0 (8.8, 15.0) | 0.10 |

Abbreviations: aIAS indicates asymptomatic intracranial atherosclerotic stenosis; PAD, parental arterial disease; mRS, modified Ranking Scale; BMI, body mass index; SBP, systolic blood pressure; DBP, diastolic blood pressure; TG, triglyceride; TC, total cholesterol; HDL-C, high-density lipoprotein cholesterol; LDL-C, low-density lipoprotein cholesterol; HbA1c, glycosylated hemoglobin; hs-CRP, high-sensitivity C-reactive protein; NIHSS, National Institutes of Health Stroke Scale; pSSI, proximal single subcortical infarction.

^*^ *p* < 0.05 was considered statistically significant.

Supplementary Table S3. Comparisons of clinical characteristics between patients divided based on the presence of aIAS and/or PAD

| Clinical characteristics | aIAS^-^PAD^-^  (n=111) | PAD^+^ only  (n=30) | aIAS^+^ only  (n=95) | aIAS^+^PAD^+^  (n=62) | P-value |
| --- | --- | --- | --- | --- | --- |
| Female, n (%) | 28 (25.2) | 12 (40.0) | 37 (38.9) | 38 (61.3) | <0.001^*^ |
| Age (year), mean ± SD | 62.7±10.8 | 63.9±8.5 | 67.7±10.5 | 69.2±10.7 | <0.001^*^ |
| Hypertension, n (%) | 62 (55.9) | 19 (63.3) | 76 (80.0) | 49 (79.0) | <0.001^*^ |
| Diabetes mellitus, n (%) | 27 (24.3) | 8 (26.7) | 40 (42.1) | 28 (45.2) | 0.01^*^ |
| Smoking, n (%) | 57 (51.4) | 15 (50.0) | 35 (36.8) | 14 (22.6) | 0.002^*^ |
| Alcohol consumption, n (%) | 48 (43.2) | 10 (33.3) | 30 (31.6) | 11 (17.7) | 0.008^*^ |
| Pre-stroke mRS (points), median (IQR) | 0 (0, 0) | 0 (0, 0) | 0 (0, 0) | 0 (0, 0) | 0.20 |
| BMI, mean ± SD | 25.51±3.25 | 24.72±2.64 | 24.98±3.53 | 24.63±3.90 | 0.35 |
| SBP (mmHg), median (IQR) | 152.0 (140.0, 167.0) | 153.0 (144.0, 166.2) | 150.0 (142.0, 162.0) | 162.0 (147.0, 174.0) | 0.11 |
| DBP (mmHg), median (IQR) | 87.0 (78.0, 95.0) | 83.5 (74.0, 90.0) | 81.0 (74.0, 92.0) | 82.5 (73.3, 91.0) | 0.073 |
| TG (mmol/L), median (IQR) | 1.42 (1.02, 2.09) | 1.50 (0.98, 2.39) | 1.46 (1.13, 2.05) | 1.42 (1.13, 1.84) | 0.97 |
| TC (mmol/L), mean ± SD | 4.66±0.96 | 4.65±1.05 | 4.55±1.01 | 4.75±1.14 | 0.70 |
| HDL-C (mmol/L), median (IQR) | 0.97 (0.83, 1.19) | 0.91 (0.79, 1.10) | 1.06 (0.86, 1.33) | 1.18 (0.93, 1.40) | 0.015^*^ |
| LDL-C (mmol/L), mean ± SD | 2.73±0.87 | 2.76±0.79 | 2.66±0.84 | 2.80±0.94 | 0.82 |
| Uric acid (mmol/L), median (IQR) | 294.00  (253.00, 371.00) | 313.00  (244.25, 423.25) | 291.50  (239.93, 359.67) | 296.50  (233.73, 401.25) | 0.66 |
| HbA1c (%), median (IQR) | 6.10 (5.80, 7.50) | 6.15 (5.80, 8.50) | 6.40 (5.80, 8.45) | 6.80 (5.80, 8.45) | 0.13 |
| Homocysteine (mmol/L), median (IQR) | 11.5 (9.3, 14.2) | 12.4 (9.5, 15.1) | 10.9 (9.0, 14.8) | 11.2 (8.9, 15.3) | 0.86 |
| Neutrophil count (×10^9^/L), median (IQR) | 4.30 (3.40, 5.40) | 4.20 (3.28, 4.95) | 4.30 (3.58, 5.70) | 4.60 (3.78, 5.88) | 0.15 |
| Hs-CRP (mg/L), median (IQR) | 0.80 (0.50, 2.20) | 1.15 (0.63, 1.70) | 0.85 (0.50, 2.32) | 1.50 (0.50, 3.60) | 0.24 |
| Initial NIHSS (point), median (IQR) | 2.0 (1.0, 3.0) | 1.5 (0.0, 3.0) | 2.0 (1.0, 3.0) | 3.0 (1.0, 5.0) | 0.005^*^ |
| Infarct size (mm), median (IQR) | 11.80 (9.74, 16.82) | 13.59 (8.73, 18.90) | 14.52 (10.63, 19.34) | 17.26 (10.36, 25.64) | 0.002^*^ |
| pSSI, n (%) | 72 (64.9) | 21 (70.0) | 65 (68.4) | 51 (82.3) | 0.11 |
| Anti-platelet, n (%) | 107 (96.4) | 28 (93.3) | 90 (94.7) | 57 (91.9) | 0.65 |
| Statins, n (%) | 110 (99.1) | 30 (100.0) | 95 (100.0) | 60 (96.8) | 0.33 |
| Posterior lesion, n (%) | 29 (26.1) | 4 (13.3) | 43 (45.3) | 21 (33.9) | 0.003^*^ |
| Hospital stay (day), median (IQR) | 10.0 (8.0, 12.0) | 11.0 (9.0, 13.0) | 10.0 (9.0, 12.0) | 12.0 (8.8, 15.0) | 0.005^*^ |

Abbreviations: aIAS indicates asymptomatic intracranial atherosclerotic stenosis; PAD, parental arterial disease; mRS, modified Ranking Scale; BMI, body mass index; BMI, body mass index; SBP, systolic blood pressure; DBP, diastolic blood pressure; TG, triglyceride; TC, total cholesterol; HDL-C, high-density lipoprotein cholesterol; LDL-C, low-density lipoprotein cholesterol; HbA1c, glycosylated hemoglobin; hs-CRP, high-sensitivity C-reactive protein; NIHSS, National Institutes of Health Stroke Scale; pSSI, proximal single subcortical infarction.

^*^ *p* < 0.05 was considered statistically significant.
